# Prevalence of clinical trial status discrepancies: A cross-sectional study of 10,492 trials registered on both ClinicalTrials.gov and the European Union Clinical Trials Register

**DOI:** 10.1371/journal.pone.0193088

**Published:** 2018-03-07

**Authors:** Jessica Fleminger, Ben Goldacre

**Affiliations:** Centre for Evidence Based Medicine, Department of Primary Care Health Sciences, University of Oxford, Oxford, United Kingdom; Karolinska Institutet, SWEDEN

## Abstract

**Objective:**

Trial registries are a key source of information for clinicians and researchers. While building OpenTrials, an open database of public trial information, we identified errors and omissions in registries, including discrepancies between descriptions of the same trial in different registries. We set out to ascertain the prevalence of discrepancies in trial completion status using a cohort of trials registered on both the European Union Clinical Trials Register (EUCTR) and ClinicalTrials.gov.

**Study design and setting:**

We used matching titles and registry IDs provided by both registries to build a cohort of dual-registered trials. Completion statuses were compared; we calculated descriptive statistics on the prevalence of discrepancies.

**Results:**

11,988 dual-registered trials were identified. 1,496 did not provide a comparable completion status, leaving 10,492 trials. 16.2% were discrepant on completion status. The majority of discrepancies (90.5%) were a ‘completed’ trial on ClinicalTrials.gov inaccurately marked as ‘ongoing’ on EUCTR. Overall, 33.9% of dual-registered trials described as 'ongoing' on EUCTR were listed as 'completed' on ClinicalTrials.gov.

**Conclusion:**

Completion status on registries is commonly inaccurate. Previous work on publication bias may underestimate non-reporting. We describe simple steps registry owners and trialists could take to improve accuracy.

## Introduction

Trial registries have been developed over the past three decades to address ongoing structural problems in evidence-based medicine including non-publication of trial results [1–3], selective reporting of results in trial publications [4], and duplication of research [5]. Trial registration is required by law for some trials in various territories including the United States (US) (Food and Drug Administration Modernization Act (FDAMA)[6], Food and Drug Administration Amendments Act (FDAAA)[6]) and the European Union and European Economic Area (European Medicines Agency (EMA)[7]). Registration is also the subject of guidance from organisations including the International Committee of Medical Journal Editors (ICMJE)[8] and the World Health Organisation (WHO)[9], which provide international standards on the types of information to be included in a clinical trial registries.

Trial registries have therefore become an important and routine source of information for clinicians and researchers seeking trials whose results should be available already, may be available on request, or whose results may be imminent. Trial registries have also become an important source of information for research assessing the extent of non-publication of completed clinical trials. The most recent systematic review on publication bias [2] summarises the results of 22 cohort studies that each take a sample of trials denoted on registries as 'complete' (or 'terminated'), and then seek to establish whether results are available. A subset of these cohort studies also use registry data on trial 'completion date' to assess publication delay, or compliance with guidance on timely reporting. However the information on trial registries must be current and accurate in order for the results of these cohort studies to be valid, and for registries to achieve their objectives. If, for example, a trial is incorrectly denoted on a registry as 'ongoing' when in reality it is 'complete' then a clinician, patient, researcher or auditor may be falsely assured that no results are due.

While building OpenTrials [10], an open database aiming to link together all publically available information from all trials, we identified various errors and omissions in the content of registries, and discrepancies between descriptions of the same trial in different registries. Dual-registered trials can provide an insight into the extent of incorrect information on registries, as a discrepancy between two registries demonstrates that the data on at least one are incorrect. We therefore set out to describe the prevalence of incorrect completion statuses on two major registries, the EU Clinical Trials Register (EUCTR) and ClinicalTrials.gov, by examining discrepancies in trial completion status and reporting status for all trials registered on both platforms.

## Methods

Using the OpenTrials database we created a dataset of all trials registered on both ClinicalTrials.gov (as of 02/06/2017) and EUCTR (as of 15/06/17). The ClinicalTrials.gov and EUCTR data available in the OpenTrials database were generated from a simple download of both registries and therefore contained all trials registered on these registries, as of the date that they were accessed. We required any one of three conditions to be met in order to classify a trial as registered on both registries: 1) the ClinicalTrials.gov ID was listed in the EUCTR entry; 2) the EUCTR ID was listed in the ClinicalTrials.gov entry; or 3) the trial title was an exact match on both registries. Trials were matched from EUCTR to ClinicalTrials.gov using the ‘US NCT (ClinicalTrials.gov registry) number’ field in EUCTR records. Trials were matched from ClinicalTrials.gov to EUCTR using the ‘Other Study ID Numbers’ field in ClinicalTrials.gov. In addition to matching using secondary IDs, we also matched trials using the text of the title (‘Official Title’ in ClinicalTrials.gov and ‘Full title of the trial’ in EUCTR). In order to limit the number of incorrect matches, we only matched trials if the full text of the title was exactly the same on the two registries. We excluded any one-to-many or many-to-many matches. As all trials registered on EUCTR are interventional clinical trials, the final dataset of matched trials only included interventional clinical trials.

Each registry uses its own terminology and definitions for ‘completion status’. On ClinicalTrials.gov we considered a trial as 'ongoing' if it had one of the following statuses: ‘Active, not recruiting’, ‘Available’, ‘Enrolling by invitation’, ‘Not yet recruiting’, ‘Recruiting’ or ‘Suspended’. We considered a trial as 'completed' if it had one of the following statuses: ‘Completed’ or ‘Terminated’. We also considered a trial as 'completed' if it had results uploaded onto ClinicalTrials.gov. We excluded trials with the following statuses on ClinicalTrials.gov: ‘Withdrawn’, ‘Approved for marketing’, ‘No longer available’, ‘Temporarily not available’ or ‘Withheld’.

On EUCTR, each trial is broken down into separate ‘protocols’ (effectively registry entries) specific to each member state. For example, if a trial is run in France, Belgium and Germany then the registry holds three protocols, each with its own completion status. There is no headline completion status that covers the entire trial and therefore the status of a trial must be inferred from the statuses of the individual states. Because of this complexity we considered a trial on EUCTR as 'completed' if *all* member state protocols had one of the following statuses: ‘Completed’ or ‘Prematurely Ended’. We also considered a trial as 'completed' if it had results uploaded onto EUCTR. We considered a trial as 'ongoing' if *any* of its underlying protocols had any of the following statuses: ‘Ongoing’, ‘Restarted’, ‘Suspended by CA’ or ‘Temporarily Halted’. We excluded trials where any of its underlying protocols had any of the following statuses: ‘Not Authorised’ or ‘Prohibited by CA’ ('Competent Authority'). We also excluded all trials registered on EUCTR which were performed entirely outside the EU/EAA, as these trials do not have any member state protocols associated with them and therefore do not provide a completion status.

Table 1 summarises the mappings we made between each registry to create a common, comparable completion status. Completion statuses were compared between the two registries and descriptive statistics for the prevalence of discrepancies were calculated. We performed a manual check of 20 randomly selected discrepant trials. As we are aware of cases where different trials within the same registry have the same title, we manually checked all discrepant trials matched on title alone, to confirm that they were descriptions of the same trial. We also manually checked the statuses of 100 discrepant trials matched on title alone, to confirm that they matched our dataset.

**Table 1:** Mapping EUCTR and ClinicalTrials.gov onto comparable completion statuses.

	Ongoing	Completed	Excluded from analysis
ClinicalTrials.gov	Active, not recruiting	Completed	No longer available
	Available	Terminated	Temporarily not available
	Enrolling by invitation		Withheld
	Not yet recruiting		Withdrawn
	Recruiting		Approved for marketing
	Suspended		
EUCTR	Ongoing	Completed	Not Authorised
	Restarted	Prematurely Ended	Prohibited by CA
	Suspended by CA		
	Temporarily Halted		

## Results

**Fig 1:**
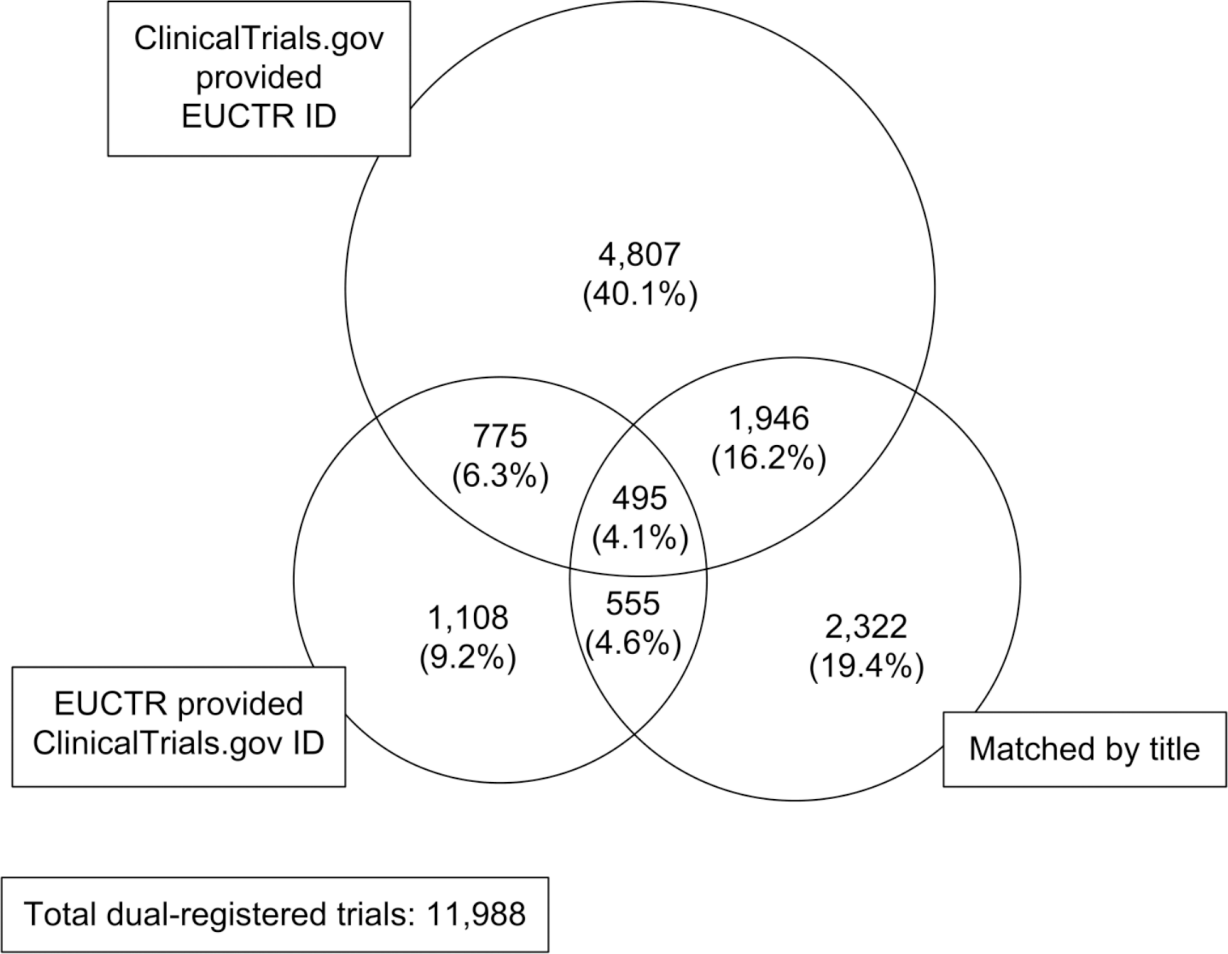
Sources of matches to generate dual-registered trials.

As at 15/06/2017 EUCTR contained 30,492 trials, 3,141 (10.3%) of which contained a ‘US NCT (ClinicalTrials.gov registry) number’. As at 02/06/2017 ClinicalTrials.gov contained 237,230 studies, 63,166 (26.6%) of which contained at least one secondary ID in ‘Other Study ID Numbers’. Matching from ClinicalTrials.gov to EUCTR using the ClinicalTrials.gov ‘Other Study ID Numbers’ data field yielded 8,003 matches. Matching from EUCTR onto ClinicalTrials.gov using the EUCTR ‘US NCT (ClinicalTrials.gov registry) number’ data field yielded 2,913 matches, of which 1,250 (43.0%) were already matched by the preceding method. Matching on title yielded 5,318 matches, of which 2,996 (56.3%) were already matched by the preceding two methods. In total this yielded a dataset of 11,988 dual-registered trials (see Fig 1). Of the 11,988 dual-registered trials, EUCTR provided a matching ClinicalTrials.gov ID for 24.3%, ClinicalTrials.gov provided a matching EUCTR ID for 66.8% and 44.4% had identical titles for matching.

Of the 11,988 dual-registered trials, 782 trials (6.5%) were excluded because EUCTR did not provide a completion status (they were undertaken entirely outside the EU/EAA). 728 trials (6.1%) were excluded because one or both of their completion statuses were neither ongoing, nor complete, but rather met our status exclusion criteria (due to the trial being, for example, withdrawn, temporarily unavailable, or rejected by the competent authority). 14 trials met both these exclusion criteria. We therefore excluded 1,496 trials in total (12.5%), leaving 10,492 dual-registered trials (87.5%) for analysis.

**Fig 2:**
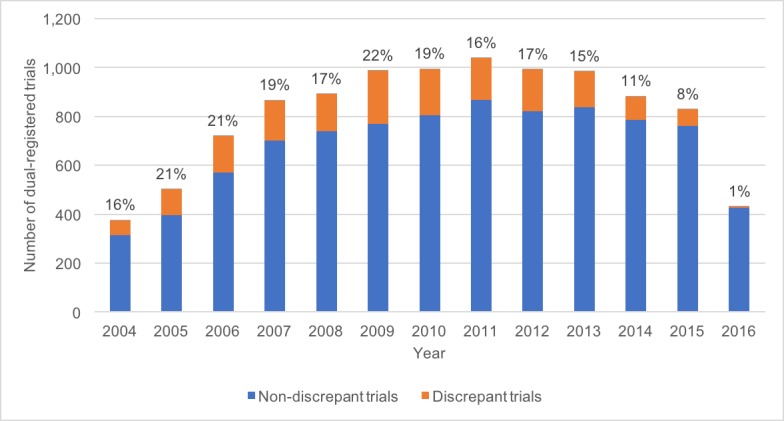
Proportion of dual-registered trials that are discrepant by year of EUCTR registration.

8,794 (83.8%) of the included dual-registered trials were not discrepant (both registries were marked ‘completed’ or both were marked ‘ongoing’) leaving 1,698 (16.2%) discrepant trials (‘completed’ on one registry and ‘ongoing’ on another). Out of the 1,698 discrepant trials, 1,536 (90.5%) were ‘ongoing’ on EUCTR and ‘complete’ on ClinicalTrials.gov, while the remainder (162, 9.5%) were ‘complete’ on EUCTR and ‘ongoing’ on ClinicalTrials.gov. The source of the discrepancy is assumed to be the registry which has failed to be updated from 'ongoing' to completed. Fig 2 shows the proportion of discrepant trials by year of EUCTR registration. We checked 100 (27.0%) of these dual-registered trials to confirm that their statuses correctly matched our data. We also performed a random manual check on the current web pages of the registry entries for 20 (1.2%) trials with status discrepancies: all 20 correctly matched our data. To ensure that all 371 discrepant trials matched between registries by title alone were indeed two registry entries for the same trial, we manually checked all 371 trials by ensuring that there were no substantial discrepancies between investigators, outcomes, disease area, locations, and number of participants. All 371 (100%) were true matches.

4,530/10,492 (43.1%) dual-registered trials were 'ongoing' on EUCTR. Out of these 'ongoing' trials, 1,536/4,530 (33.9%) were marked as 'complete' on ClinicalTrials.gov. Therefore 33.9% of trials described as 'ongoing' on EUCTR were in reality 'completed'. Conversely 3,156/10,492 (30.0%) dual-registered trials were 'ongoing' on ClinicalTrials.gov: of these, 162/3,156 (5.1%) were marked as 'complete' on EUCTR (Table 2). The median time since commencement for discrepant trials was 9 years; and for concordant trials was 7 years.

**Table 2:** Discrepancies in completion status in 'ongoing' dual registered trials.

	EUCTR	ClinicalTrials.gov
Total 'ongoing' trials	4,530	3,156
'Ongoing' trials marked 'completed' on other registry	1,536 (33.9%)	162 (5.1%)

## Discussion

In a dataset of 10,492 dual registered trials, we found that 16.2% were discrepant on completion status. The majority of these discrepancies (90.5%) were due to a trial being described as 'completed' on ClinicalTrials.gov, but still marked as 'ongoing' on EUCTR; since a registry entry is manually changed to ‘completed’, this suggests that the data on EUCTR are more commonly incorrect. Of the dual-registered trials that were 'ongoing' on EUCTR, 33.9% were 'complete' on ClinicalTrials.gov; therefore in a third of cases the status appears to be incorrect, and the positive predictive value (the likelihood that a trial marked as 'ongoing' is actually 'ongoing') is only 66.1% for EUCTR. By comparison, only 5.1% of the dual-registered trials marked as 'ongoing' on ClinicalTrials.gov were 'complete' on EUCTR, making the positive predictive value 94.9% for ClinicalTrials.gov.

### Strengths and weaknesses

To our knowledge this is the first study to examine discrepancies between registries. We were able to utilise the OpenTrials database to match dual-registered trials and compare their statuses on the two different registries. This enabled us to analyse all registered trials, rather than limiting the analysis to a smaller manual sample of dual-registered trials. The two week time delay between the collection of data on EUCTR and ClinicalTrials.gov could have slightly inflated the number of discrepancies between the two registries: however this could not realistically explain the extremely high prevalence of apparently incorrect statuses on EUCTR; and in our manual check of the live registry entries (20 randomly selected trials and all 371 trials matched by title alone where we identified discrepancies using our extracted data) our data were correct about the discrepancy. Our matches were based on reciprocal IDs being provided by each registry, and by identical title. This matching strategy is conservative: specifically, it is very likely that we missed dual-registered trials where the researchers either failed to provide an existing additional registry ID in one of their registry entries, or because the official titles that they used on the two registries differed. We only analysed trials that were registered on ClinicalTrials.gov and EUCTR: there are dozens of trial registries globally and discrepancy rates between registries may vary, especially if some registries are viewed by trialists as more important than others. By including trials that commenced recently, for example in 2016, it is possible we have underestimated the prevalence of trial status discrepancies, as very recently commenced trials may not have yet had the opportunity to complete and so develop a discrepant trial completion status; however to actively exclude these trials would also risk excluding genuinely completed trials for which data exist and which are at genuine risk of having a discrepant status; therefore on balance we have concluded it is best to include all trials currently on the registries.

We believe that our findings are likely to generalise to all trials, and that completion status data are likely to also be unreliable for single-registered trials. Specifically, we do not believe that there is any systematic difference between dual-registered trials, and trials registered on one register, that would lead the former to be more susceptible to having an inaccurate completion status. Indeed, the opposite may be true: dual-registered trials may, for example, be more likely to be multinational trials, larger than average, and more likely to have external funding; large trials may be expected to have a larger and more professional administrative team focused on compliance issues; and there is evidence that industry-funded trials are more likely to comply with registration requirements [11]; however there is no prior work on how trial size may impact the accuracy of registry data, and work on publication bias has shown no relationship between results reporting and sample size[12]. In addition, there is no database that could be used to assess whether the completion status is correct for a trial registered on one register. This could therefore only be assessed by contacting trialists directly: a laborious research project, where response rates may be poor.

### Context of other findings

We are aware of no previous research specifically exploring discrepancies in trial registry entries, and only one publication on errors in registry entries: a 2017 cohort study which found that 34/92 (37.0%) trials listed as “ongoing” on their registry entry also had a publication containing results of the completed trial [13]. There is some work on completeness of registry entries [14], which found 224 missing registry fields (from the WHO Minimum Data Set [9]) in 152 RCTs registered on ClinicalTrials.gov. The most commonly empty field was ‘key secondary outcomes’ (44.1% of RCTs) followed by ‘primary outcome’ (34.1% of RCTs).

### Policy implications and further research

We found that the completion status given on trial registry entries is commonly incorrect. This has a number of concerning implications. Trial completion status is used by researchers in publication bias cohort studies to identify trials where results are due. If trials are incorrectly denoted as ongoing, when in reality they are completed, then they will be excluded from publication bias cohorts: if trials with incorrect status are also more likely to be unreported (which is possible, as the trialists are not competently maintaining their registry data) then this would mean that cohorts in studies of publication bias will exhibit selection bias, leading to underestimates of non-publication. In addition, systematic reviewers commonly search on registries for only completed trials, and then seek results for these studies: our results strongly suggest that completion status is sufficiently unreliable that systematic reviewers should include ongoing trials from registries in their search strategy.

It is unclear whether responsibility for the errors identified lies with researchers, registry owners, or both. The differential error rate between EUCTR and ClinicalTrials.gov is striking, and suggests that either trialists consistently regard updating EUCTR data as a lower priority, or that updates given to EUCTR by trialists are not passed on to the public registry. We would welcome clarification on this from registry owners, and suggest that registry owners should have simple automated data checks in place. Where discrepancies have been identified they should be flagged on registries; where discrepancies on key data fields are identified for dual-registered trials, the trialists should be contacted and asked for clarification; and where clarification has been requested but none given, this should also be noted publicly on the registry. We also suggest that trialists should give higher priority to ensuring their registry data are accurate and current.

## Conclusions

Trial completion status on registries is commonly inaccurate. 33.9% of dual-registered trials listed as 'ongoing' on EUCTR are listed as 'completed' on ClinicalTrials.gov. Previous work on publication bias may have underestimated the rate of non-reporting. Registry owners should undertake simple cross-checks of data to ensure completion status is accurate.
